# Monoclonal gammopathy of renal significance (MGRS): retrospective monocentric analysis of clinical outcomes and treatment strategies

**DOI:** 10.1007/s10238-025-01646-7

**Published:** 2025-04-15

**Authors:** Pasquale Esposito, Lucia Macciò, Antonia Cagnetta, Francesca Costigliolo, Emilio Venturelli, Elisa Russo, Marco Gallo, Debora Soncini, Francesca Viazzi, Roberto Massimo Lemoli, Michele Cea

**Affiliations:** 1 Unit of Nephrology, Dialysis and Transplantation Department of Internal Medicine and Medical Specialties (DIMI), University of Genoa, Genoa, Italy; 2 IRCCS Ospedale Policlinico San Martino, Genoa, Italy; 3 Clinic of Hematology, Department of Internal Medicine and Medical Specialties (DiMI), University of Genoa, Genoa, Italy

**Keywords:** Monoclonal gammopathy of renal significance, Amyloidosis, Kidney, Anemia, Proteinuria, Bone marrow plasma cells

## Abstract

Monoclonal Gammopathy of Renal Significance (MGRS) is a group of rare disorders in which monoclonal proteins cause kidney damage. Due to its rarity, ongoing research is vital to refine diagnostics, enhance treatment, and improve outcomes. This retrospective study analyzed 34 patients with renal biopsy-proven MGRS-defining lesions. Patients were divided into two subgroups: kidney-limited AL amyloidosis (MGRS-A, 44%, n = 15) and other MGRS (MGRS-NA, 56%, n = 19). Key outcomes included progression-free survival and overall survival. Baseline characteristics such as histopathology, plasma cell percentage, kidney function, and proteinuria were documented alongside initial treatments, and hematologic and renal response. Distinct differences were observed between the two groups: MGRS-NA was primarily associated with glomerular lesions, while MGRS-A exhibited broader kidney involvement. Treatment varied: bortezomib for plasma cell-driven cases and rituximab for B-cell-related conditions. Anemia was the most common side effect (71%), associated with treatment intensity. Despite similar overall survival outcomes, MGRS-A followed a more aggressive course, with a shorter time from diagnosis to death (206 vs. 728 days). Renal and hematologic responses were comparable between the groups, although baseline factors such as hemoglobin and CRP levels were predictive of mortality. These findings underscore the need for more precise characterization and standardized criteria to optimize the management of MGRS.

**Supplementary Information:**

The online version contains supplementary material available at 10.1007/s10238-025-01646-7.

## Introduction

Monoclonal gammopathies (MG) are uncommon clinical conditions often identified through laboratory tests, particularly in elderly individuals [1]. These disorders are defined by the presence of monoclonal immunoglobulin (Ig) in plasma, urine, or both. Contemporary hematological classification criteria highlight the importance of identifying the pathological cell clone and evaluating the extent of associated organ damage [2]. When patients meet diagnostic criteria for neoplasia but exhibit no organ damage, they may be classified with “smoldering” conditions, such as smoldering multiple myeloma (SMM), low-grade chronic lymphocytic leukemia (CLL), or smoldering lymphoplasmacytic lymphoma (sLPL). In cases where both tumor burden and organ damage are minimal or absent, diagnoses may include monoclonal gammopathy of undetermined significance (MGUS), IgM-MGUS, or monoclonal B lymphocytosis, conditions often linked to lymphoplasmacytic lymphoma or CLL clones [2]. MGUS was first described by Robert Kyle in 1978, representing a pivotal milestone in the classification and understanding of these disorders [3, 4]. Since its identification, MGUS has been widely recognized as a precursor to more serious plasma cell dyscrasias, such as multiple myeloma, and remains a focus of active research into its progression and clinical implications. Traditionally, monoclonal immunoglobulin (Ig) has been associated with renal disease in overtly malignant conditions, such as multiple myeloma (MM) or lymphoplasmacytic lymphoma. However, recent evidence indicates that monoclonal Ig can also cause kidney damage in the absence of a significant tumor burden, challenging long-standing assumptions about the pathophysiology of these conditions [2–4]. Given the renal risks associated with nephrotoxic monoclonal Ig in these cases, the International Kidney and Monoclonal Gammopathy Research Group (IKMG) introduced the term “Monoclonal Gammopathy of Renal Significance” (MGRS) in 2012, later updating it in 2017. MGRS refers to clonal plasma cell or B-cell disorders that produce monoclonal Ig capable of causing renal damage, even in the absence of cancer treatment criteria [2–4]. This classification emphasized the importance of timely therapeutic intervention to prevent end-stage renal disease in MGRS patients. If MGRS progresses to malignancies such as multiple myeloma or high-grade CLL, standard oncologic treatments are typically recommended. The prevalence of MGRS remains uncertain due to its recent classification, with estimates ranging from 1.5 to 6% of MGUS patients [5]–8. Kidney damage associated with MGRS is particularly resistant to standard immunosuppressive therapies, with a recurrence rate as high as 90% following kidney transplantation if the monoclonal gammopathy remains uncontrolled [9–11]. The situation is further complicated by the potential coexistence of MGRS with conditions like amyloidosis, a group of diseases characterized by the accumulation of insoluble protein aggregates in a beta-sheet configuration. Among these, immunoglobulin light chain amyloidosis (AL) is the most common and frequently occurs alongside MG, complicating patient management. Systemic AL amyloidosis affects approximately 10% of MM patients and significantly influences prognosis, particularly in cases with cardiac involvement.

Diagnosing this form of amyloidosis requires precision, typically achieved through Congo Red staining, immunohistochemistry, or electron microscopy to confirm the presence of amyloid deposits in affected organs. Additionally, mass spectrometry can be employed for amyloid typing [12, 13].

Advancements in treatment, including the addition of the anti-CD38 antibody daratumumab, have expanded therapeutic options for AL amyloidosis, highlighting the importance of thorough characterization of MG patients. Recent studies in the context of MGRS have revealed variations in clinical outcomes based on amyloidosis involvement. For instance, a study by Gozzetti et al. [14], involving 280 patients, found that MGRS patients without amyloidosis (MGRS-NA) had more severe renal impairment compared to those with amyloidosis (MGRS-A). However, MGRS-A patients exhibited lower overall survival, likely due to the impact of cardiac involvement. These findings suggest that while amyloidosis adds complexity to MGRS, its effects extend beyond renal impairment and influence survival through involvement of other organs. To address these complexities in real-life setting, we conducted a retrospective study to compare the clinical and histological presentations between MGRS-NA and kidney-limited MGRS-A patients. Additionally, we analyzed various treatment approaches, focusing on hematologic and renal responses, as well as overall prognosis. This investigation aims to enhance our understanding of MGRS subtypes, inform therapeutic strategies, and identify predictive markers for improved outcomes.

## Materials and methods

This observational, retrospective and monocentric study involved adult patients (over 18 years old) diagnosed with MGRS according to the IKMG criteria, [4] at Polyclinic San Martino Hospital in Genoa, Italy. Specifically, all patients were classified into MGRS-NA and MGRS-A groups, based on the Congo Red positivity of their kidney biopsies. A bone marrow (BM) biopsy was performed to screen the presence of tumor cells clone and to exclude a MM diagnosis; only patients without clinical signs of overt MM were included in the study. In contrast, those with systemic involvement, such as neurological or cardiac complications, were excluded. Specifically, any features of cardiac involvement identified through echocardiography, such as unexplained left ventricular wall thickening, diastolic dysfunction, or reduced global longitudinal left ventricular strain, were considered exclusion criteria. Clinical and laboratory data were collected from the electronic medical record system. At diagnosis, the following parameters were analyzed: age, sex, performance status (evaluated by the Eastern Cooperative Oncology Group score-ECOG), renal function, including serum creatinine (sCr) and estimated glomerular filtration rate (eGFR), proteinuria, hemoglobin level, serum calcium and albumin, beta-2-microglobulin, lactate dehydrogenase (LDH), immunoglobulin-specific isotype, serum-free light-chain (sFLC), percentage of monoclonal bone marrow plasma cells, and the presence of B-cell lymphoproliferative disease. eGFR was calculated using the Chronic Kidney Disease Epidemiology Collaboration (CKD-EPI) creatinine-based equation [15]. Additional variables included the Pavia renal staging system, the International Staging System score (ISS), and frontline treatments [16]. The primary endpoint was to evaluate hematologic and renal responses, progression-free survival (PFS), time to next treatment (TNT), and overall survival (OS). Secondary endpoints included a description of baseline patient characteristics, the most frequently used therapies, the relationship between hematologic and renal responses, and the safety and tolerability of treatments. Hematologic responses were defined according to the International Myeloma Working Group (IMWG) criteria [17, 18]. Renal response was defined as a reduction in 24 h urine protein output by at least 30% or to less than 0.5 g/24 h, in the absence of renal progression (eGFR reduction of more than 25% from baseline). In cases where kidney biopsy slides were available, we further analyzed the distribution of primary lesions (tubular, glomerular, vascular, or mesangial) and immunofluorescence (IF) patterns, focusing on the deposition of light chains, heavy chains, and complement proteins. Each patient’s comorbidities were quantified using the age-adjusted Charlson Comorbidity Index (CCI). Follow-up visits were scheduled based on clinical judgment, with the end of follow-up defined as the last recorded observation (as of December 31, 2023), death, or disease progression. The study was conducted under all national and international ethical and legal recommendations, following approval by the local Ethics Review Committee, in accordance with the declaration of Helsinki. (Comitato Etico Territoriale, CER- Liguria: 515/2020). Informed consent was obtained from all participants prior to their inclusion in the study.

### Statistical analysis

Data were collected in spreadsheets and analyzed using R statistical software (v. 4.3.3; RStudio) and SPSS (v. 25; IBM). A *p*-value < 0.05 was considered statistically significant. For patients’ characteristics, continuous variables were expressed as mean or median and compared with the Kolmogorov–Smirnov or Student’s t-test as appropriate after Shapiro test. Categorical variables were expressed as counts and percentages and compared using the Chi-square, binomial test or Fisher’s exact test as appropriate. Survival analysis between groups was performed using the log-rank (Cochran–Mantel–Haenszel) test. The effects of individual variables on survival curves were assessed using a univariate Cox proportional hazards analysis, given the relatively small number of available cases. For each variable, a log-rank test was performed, and the corresponding p-value was reported in the variable header. To identify significant variables, Bonferroni post hoc correction was applied to the p-values, with the results included in the graph.

## Results

### Baseline characteristics of cohort

A total of 34 patients were enrolled between December 2012 and August 2021 at our center. Table 1 summarizes the demographic and clinical characteristics of the cohort, divided into two subgroups based on the histopathological features of renal biopsy: patients without amyloid deposits (MGRS-NA, 56%, n = 19) and those with amyloid tissue deposits (MGRS-A, 44%, n = 15). The mean age at diagnosis was 61.2 years (range 31–84), with no significant differences between the two groups. No differences were also observed in performance status, monoclonal protein (*p* = 0.49), ISS stage (*p* = 0.24), monoclonal protein size (*p* = 0.59), serum albumin (*p* = 0.43) and Pavia renal staging (*p* = 0.1961). By contrast, the light chains ratio (sFLC K/λ) was significantly lower in MGRS-A than in MGRS NA group (*p* < 0.001).

**Table 1:** Patients’ characteristics

Variable	All cohort	MGRS-NA	MGRS-A	*p-value* NA vs A
N (%)	34	19 (56%)	15 (44%)	
Age at diagnosis (years)	61.2 (31–84)	61 (31–84)	61.75 (61–80)	0.30**
Sex (M;F)	22;12	13;6	10;5	1.00*
*Performance status*				
Charlson Comorbidity (≤ 3; > 3)	12;22	7;12	4;11	1.00*
ECOG 0/1/2/3	10/14/8/2	5/10/4/0	5/5/4/1	0.69*
Dialysis (%)	10 (29.4)	7 (36.8)	3 (20)	0.28*
*Monoclonal protein subtype*				
IgG (%)	16 (47)	9 (47.3)	7 (46.6)	0.49*
IgA (%)	5 (14.7)	2 (15.5)	3 (20)	
IgM (%)	8 (23.5)	6 (31.5)	2 (13.36)	
Micromolecular (%)	5 (14.7)	2 (10.5)	3 (20)	
k-light chain (%)	12 (35.2)	11 (57.9)	1 (6.6)	0.003*
λ-light chain (%)	22 (64.8)	8 (42.1)	14 (93.4)	
*Disease stage*				
ISS 1;2;3	10; 9; 15	2; 6;11	8;3;4	0.24*
Bone Marrow plasma cells (%)	4.78 (0–18)	1.89 (0–18)	22 (1–15)	< 0.0001**
*Biochemical markers*				
sFLC k (mg/L)	82.19 (9.3–518)	117 (10.4–518)	37.7 (9.3–106)	0.01**
sFLC λ (mg/L)	125.85 (11.1–844)	74.2 (14.9–200)	191 (11.1–844)	0.14**
sFLC k/ λ ratio	1.26 (0.01–8.2)	1.89 (0.55–8.2)	0.474 (0.01–2.02)	< 0.001**
β2 microglobulin (mg/L)	5.8 (1.6–18.1)	6.49 (1.6–12.2)	5.12 (1.8–18.1)	0.04**
Monoclonal protein (g/L)	4.85 (0–33.5)	3.52 (0–17.4)	6.54 (0–33.5)	0.59**
LDH (U/L)	234 (153–493)	252 (153–493)	212 (158–319)	0.04**
Hemoglobin (g/dl)	11.1 (7.8–15.7)	10.3 (7.8–13.3)	12.3 (8.8–15.7)	0.02**
Albumin (g/dl)	3 (1.12–4.29)	3.04 (1.12–4.14)	2.95 (1.7–4.3)	0.43**
CRP (mg/dl)	19.75 (0- 121)	21,24 (0–79.3)	17.6 (0–121)	0.30**
sCr (mg/dl)	2.49 (0.6–7.8)	3.16 (0.7–7.88)	1.64 (0.6–4.7)	0.01**
eGFR (ml/min)	44.6 (5–110)	33 (5–95)	59.3 (11–110)	0.04**
Proteinuria 24 h (g/24 h)	3.96 (0.11–10)	3.56 (0.11–10)	4.49 (0.36–8.8)	0.30**
*Pavia renal staging*				
Stage 1 (%)	10 (29.4)	3 (15.7)	7 (46.6)	0.19*
Stage 2 (%)	16 (47)	11 (58.8)	5 (33.3)	
Stage 3 (%)	8 (23.6)	5 (26.3)	3 (20)	

Bold value indicates statistically significant results

*MGRS* Monoclonal Gammopathy of Renal Significance, *NA* Non-Amyloidosis, A Amyloidosis, *ECOG* Eastern Cooperative Oncology Group performance status, *ISS* International Staging System, *FLC* Free Light Chain, *ISS* International Staging System, *LDH* Lactate Dehydrogenase, *CRP* C-reactive protein, sCr serum Creatinine, eGFR estimated Glomerular Filtration Rate. *Shapiro test, ** Kolmogorov Smirnov test

Additionally, monoclonal gammopathy-defining features, such as LDH (237 vs. 235 U/L; *p* = 0.0429) and β2-microglobulin (5.69 vs. 5.7 mg/L; *p* = 0.0423), were significantly elevated in the MGRS-A group. In contrast, patients in the MGRS-A group had less severe hematological and renal impairments, as evidenced by significantly higher hemoglobin levels (11 vs. 10.98 g/dL; *p* = 0.023), lower sCr levels (2.4 vs. 2.5 mg/dL;* p* = 0.0149), and higher eGFR (46 vs. 45 mL/min/1.73m^2^; *p* = 0.04). No significant differences were found between the groups in 24-h urine protein output (4.44 vs. 3.83 g/24 h; *p* = 0.30) or Pavia renal staging (*p* = 0.19). Interestingly, our analysis, in contrast to previously reported data, [14] revealed significant differences in the bone marrow monoclonal plasma cell percentage, with the MGRS-A group exhibiting a significantly higher percentage than the MGRS-NA group (22% vs. 1.89%; p < 0.0001).

### Renal histology

To further investigate renal impairment in our cohort, we analyzed the available histopathological data from 29 subjects. Notably, five patients (2 in MGRS-NA and 3 in MGRS-A group) were excluded from this analysis as their kidney biopsies and histological diagnoses were performed at external centers, where the slides were unavailable for critical reevaluation.

As expected, MGRS-NA encompassed patients with heterogeneous histological patterns. Among them, the majority (11 out of 17, 64.7%) were diagnosed with proliferative glomerulonephritis with monoclonal IgG deposits (PGNMID).

As shown in Table 2, glomerular involvement was predominant (82.7%) across both groups MGRS-NA and MGRS-A as well. In detail, patients in MGRS-NA group exhibited primarily glomerular lesions, with tubular and vascular involvement observed only in a few cases: 6 and 2, respectively. This pattern suggests a specific renal pathology profile for this group in which glomerular lesions are predominant. Conversely, in the MGRS-A group, tubule-interstitial and vascular lesions were more frequently observed, affecting 75% and 66.7% of patients, respectively. Notably, these differences between the two groups were statistically significant, with p-values of 0.035 for tubule-interstitial lesions and 0.002 for vascular lesions. These findings indicate a more extensive renal involvement in the amyloid group compared to MGRS-NA.

**Table 2:** Renal histology

Kidney biopsy n (%)	All cases (N = 29)	MGRS-NA (N = 17)	MGRS-A (N = 12)	p-value NA vs A*
*Optical microscopy*				
Glomerular involvement	24 (82.7)	14 (82.3)	10 (83.3)	0.94
Tubular involvement	15 (51.7)	6 (35.3)	9 (75)	**0.03**
Vascular involvement	10 (34.5)	2 (11.8)	8 (66.7)	**0.002**
IF (prevalent, + + / + + +)				
*Heavy chains*				
IgA	2 (6.9)	2 (11.8)	0 (0)	0.2
IgG	11 (37.9)	9 (52.9)	5 (41.6)	0.7
IgM	5 (17.24)	4 (23.5)	1 (8.3)	0.29
*Light chains*				
κ	7 (26.9)	6 (42.8)	1 (8.3)	**0.04**
λ	14 (53.8)	5 (35.7)	9 (75)	**0.04**
C3	10 (34.5)	9 (52.9)	1 (8.3)	**0.01**

Bold value indicates statistically significant results

*MGRS* Monoclonal Gammopathy of Renal Significance, *NA* Non-Amyloidosis, A Amyloidosis, *IF* Immunofluorescence. *Fisher test

Immunofluorescence analysis further revealed distinct immunopathological profiles between the two groups. While no statistically significant differences were observed in immunoglobulin heavy chain depositions, C3 and kappa light chain deposits were predominantly found in MGRS-NA (*p* = 0.013 and *p* = 0.04, respectively). Conversely, the MGRS-A group exhibited an almost exclusive deposition of lambda light chains, a finding that was statistically significant (*p* = 0.045).

A more detailed description of the specific histological diagnoses, lesions, and immune/amyloid deposits is available in the supplementary materials (Supplementary Table 1 for MGRS-NA and Supplementary Table 2 for MGRS-A).

### MGRS treatment strategies and side effects

All patients were treated with clone-directed therapies as first-line treatments. Bortezomib-based regimens were administered to 47% of the MGRS-NA group and 60% of the MGRS-A group, including VCD (Bortezomib, Cyclophosphamide, and Dexamethasone), VD (Bortezomib and Dexamethasone), and VMD/VMP (Bortezomib, Melphalan, and Dexamethasone/Prednisone). Rituximab-based regimens, alone or with Bendamustine, were used for 37% of the MGRS-NA group, while no patients in the MGRS-A group received anti-CD20 therapies. A small subset of patients received the anti-CD38 antibody Daratumumab (e.g., Dara-VMP or Dara-VP) or other immunomodulatory drugs, such as mycophenolate with prednisone or cyclophosphamide (see Table 3 for details). Both groups had equal rates of autologous stem cell transplantation (ASCT). Only 29.5% required second-line therapies, with three progressing to third-line treatments due to inadequate responses. Bortezomib-based regimens were preferred in MGRS-A, while Rituximab-based therapies were more common in MGRS-NA (*p* = 0.002). Toxicity analyses revealed anemia as the most frequent side effect (71%), particularly in MGRS-NA (47% vs. 24%), likely due to more intensive regimens. Other side effects included thrombocytopenia (21%), neutropenia (15%), infections (18%), cardiovascular complications (18%), and neuropathy (15%). These findings suggest a manageable safety profile, though careful monitoring was essential, especially for ASCT patients (see Table 4).

**Table 3:** Types of treatments

First LOT (n, %)	All cohort	MGRS-NA	MGRS-A	*p-value* NA vs A *
Bortezomib-*based*	18 (52.9)	9 (47.3)	9 (60)	0.002
Rituximab-*based*	7 (20.5)	7 (36.8)	0 (0)	
Daratumumab-*based*	5 (14.7)	0 (0)	5 (33.3)	
Other	3 (8.8)	3 (15.7)	1 (8)	
ASCT	6 (17.6)	3 (15.7)	3 (20)	1
*Second LOT*				
Bortezomib-*based*	2 (5.8)	1 (5.2)	1 (6.6)	
Rituximab-*based*	4 (11.7)	1(5.2)	3 (20)	
Daratumumab-*based*	1 (2.9)	0 (0)	1 (6.6)	
Other	4 (11.7)	2 (10.5)	1 (6.6)	
None	24 (70.5)	13 (68.4)	11 (73.3)	
*More advanced LOT*				
Bortezomib-*based*	0 (0)	0 (0)	0 (0)	
Rituximab-*based*	0 (0)	0 (0)	0 (0)	
Daratumumab-*based*	2 (5.8)	1 (5.2)	1 (6.6)	
Other	1 (2.9)	0 (0)	1 (6.6)	

*MGRS* Monoclonal Gammopathy of Renal Significance, *NA* Non-Amyloidosis, A Amyloidosis, *LOT* Lines of Treatments, *ASCT* Autologous Stem Cells Transplantation. *Fisher test

**Table 4:** Drug-associated toxicities

Adverse Events (n, %)	All cohort	MGRS-NA	MGRS-A	*p-value* NA vs A*
Anemia	24 (71)	16 (47)	8 (24)	0.29
Thrombocytopenia	7 (21)	5 (15)	2 (6)	
Neutropenia	5 (15)	3 (9)	2 (6)	
Infections	6 (18)	3 (9)	3 (9)	
Adverse CV events	6 (18)	2 (6)	3 (9)	
Neuropathy	5 (15)	1 (3)	5 (15)	

*MGRS* Monoclonal Gammopathy of Renal Significance, *NA* Non-Amyloidosis, A Amyloidosis, *CV* Cardio-Vascular *Fisher test

In summary, the toxicity analyses reflect the complexity of therapeutic options available for these patients, emphasizing the need for meticulous management, especially for those receiving more intensive treatments.

### Clinical outcomes

**Fig. 1:**
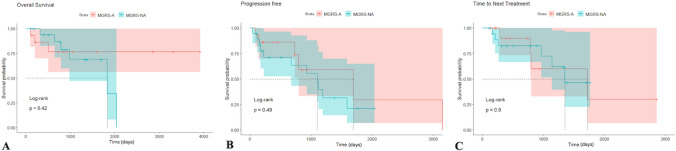
Comparisons between MGRS-NA and MGRS-A groups in term of prognosis. Kaplan-Meyer curves of the overall survival **A**, progression-free survival **B** and time to next treatment  **C** probability among MGRS-NA (blue line) or MGRS-A (red line) groups. Log-rank test is used to compute the p-value (Log-Rank test). MGRS (Monoclonal Gammopathy of Renal Significance), A (Amyloidosis), NA (Non-Amyloidosis)

**Fig. 2:**
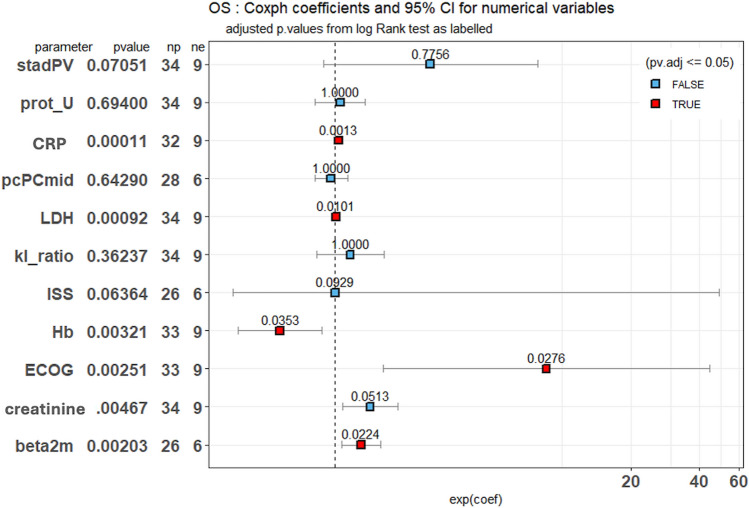
Baseline clinical and laboratory parameters and mortality risk in MGRS patients. Forest plot based on Cox proportional Hazard analysis of the indicated variables for OS in our cohort. The hazard ratios (squares) are displayed with their corresponding confidence intervals (bars) and the p-values (after post hoc correction). Significance levels are clearly indicated by both the number and color (red and blue indicating statistically significant or not, respectively). The original p-value is shown next to each analyzed parameter. Abbreviations: OS (Overall Survival), stadPV (Pavia Renal Staging), prot_U (proteinuria), CRP (C-reactive protein), pcPCmid (Medullary Plasma Cell), LDH (Lactate Dehydrogenase), ISS (International Staging System), Hb (Hemoglobin), ECOG (Eastern Cooperative Oncology Group performance status), beta2m (β2 microglobulin)

After a mean follow-up period of 33 months, 9 patients (28.1%) died, with mortality rates similar across both groups. However, patients diagnosed with MGRS-A experienced a significantly shorter interval from disease onset to death compared to those in the MGRS-NA group (206 vs. 728 days, *p* = 0.07), suggesting a more rapid disease course in the amyloid group. No significant differences were observed in OS, PFS, or time to next treatment (TNT), as shown by Kaplan–Meier analyses (Table 5 and Fig. 1). Following first-line treatment, 21 patients (61.7%) achieved a renal response, with no differences between groups regarding sCr, eGFR, or proteinuria trends. These findings indicate a comparable renal response after treatment regardless of the specific type of renal damage. In terms of hematologic responses across the cohort, 8 patients (23.5%) achieved a complete response (CR), 6 (17.6%) attained a very good partial response (VGPR), 9 (26.4%) achieved a partial response (PR), 7 (20.5%) had stable disease (SD), and 1 patient (2.9%) experienced disease progression (PD). Importantly, although hematologic response rates did not differ significantly between the two groups, the CR rate was higher in the MGRS-NA group (26.3% vs. 20%). Consequently, the entire cohort was screened to identify parameters significantly associated with mortality (Fig. 2). A multiple univariate Cox-PH analysis focusing on blood parameters at diagnosis revealed that hemoglobin level was the only parameter associated with improved OS in our cohort. By contrast, increasing levels of LDH, beta-2 microglobulin, and C-reactive protein (CRP) were significantly associated with poorer clinical outcomes. Similarly, the ECOG performance status at diagnosis showed a notable significant correlation, indicating that poor functional status also negatively impacts patient’s prognosis. Overall, these findings highlight that baseline clinical and laboratory parameters significantly influence the prognosis of MGRS patients, underscoring their potential as innovative disease biomarkers.

**Table 5:** Clinical outcomes and Responses

Event, n (%)	All cohort	MGRS-NA	MGRS-A	*p-value* NA vs A
Death, n (%)	9 (28.1)	6 (31.6)	3 (20)	
Disease onset – death interval, days (range)	516 (174–890)	728 (275–1243)	206 (103–516)	0.07*
OS, n (%)	9 (28.1)	6 (31.6)	3 (20)	
Mean, days (range)	825 (103–2039)	1101 (320–2039)	271 (103–505)	0.1*
PFS, n (%)	17 (50)	11 (58)	6 (40)	
Mean, days (range)	820 (41–3152)	667 (41–1588)	1101 (92–3152)	0.65*
TNT, n (%)	10 (6)	6 (32)	4 (27)	
Mean, days (range)	768 (162–1721)	682 (162–1349)	896 (282–1721)	0.55*
*Renal responses*				
Yes	21 (61.7)	13 (68.4)	8 (53.3)	0.47**
No	11 (32.3)	5 (26.3)	6 (40)	
Missing (no data)	2 (5.8)	1 (5.2)	1 (6.6)	
*Hematological responses*				
CR	8 (23.5)	5 (26.3)	3 (20)	0.09**
VGPR	6 (17.6)	1 (5.2)	5 (33.3)	
PR	9 (26.4)	4 (21)	5 (33.3)	
SD	7 (20.5)	6 (31.5)	1 (6.6)	
PD	1 (2.9)	1 (5.2)	0 (0)	
Missing	3 (8.8)	2 (10.5)	1 (6.6)	

*MGR*S Monoclonal Gammopathy of Renal Significance, *NA* Non-Amyloidosis, A Amyloidosis, *OS* Overall Survival, *PFS* Progression Free Survival, *TNT* Time to Next Treatment, *CR* Complete Response, *VGPR* Very Good Partial Response, *PR* Partial Response, *SD* Stable Disease, *PD* Progressive Disease. * Kolmogorov Smirnov test, ** Fisher test

## Discussion

MGRS represents a novel entity observed among kidney disease patients, particularly those presenting with substantial proteinuria. Importantly, it is not a single disease but a spectrum of conditions, each characterized by specific pathogenetic mechanisms, clinical presentations, and histological features. This heterogeneity significantly influences the clinical progression and prognosis of the disease. In this study, we explored differences among MGRS patients, focusing on the presence or absence of amyloid deposits in renal tissue. While systemic amyloidosis AL is associated with poor prognosis, especially in cases with cardiac involvement, the outcomes of kidney-restricted amyloidosis remain undefined. Our findings in MGRS-A patients align with existing data, showing a higher prevalence of clonal plasma cells producing lambda light chains compared to those producing kappa chains or full immunoglobulins/heavy chains [1]. Histologically, we observed extensive renal compartment involvement in MGRS-A patients, with light chain deposits affecting glomeruli, blood vessels, and interstitial regions, as previously reported [19]. In contrast, MGRS-NA patients exhibited IgG deposits in glomeruli and mesangial areas, consistent with findings described by Bridoux et al. [20] Interestingly, despite the distinct kidney involvement observed in MGRS-A and MGRS-NA patients, no significant differences were found in proteinuria levels between the groups. However, we noted higher creatinine and lower eGFR levels in MGRS-NA patients, consistent with poorer renal outcomes reported in previous studies [14]. All patients in our cohort were treated following current international guidelines. Specifically, the presence of IgM monoclonal proteins led to the use of Rituximab-based regimens due to their effectiveness in targeting IgM-producing B-cells [4, 5]. Patients with clonal plasma cells producing other paraproteins were treated with Bortezomib-based regimens, given their efficacy in plasma cell dyscrasias. These findings underscore the importance of accurate histopathological characterization in MGRS for selecting the most appropriate therapeutic options. Tailored treatment approaches not only improve clinical outcomes but also help preserve renal function over time. Despite distinct clinical and histological profiles in our cohort, overall outcomes were comparable between the groups. Similarly, Mancuso et al., recently reported comparable survival outcomes for patients with AL amyloidosis and other MGRS in a larger nationwide cohort, despite differences in clinical presentation [21]. Although the small sample size may have introduced some bias, our analysis reinforces previous findings, supporting the notion that kidney-limited amyloidosis does not independently worsen prognosis in MGRS patients, unlike systemic forms, which are consistently linked to poor outcomes. To further investigate factors influencing prognosis, we expanded our analysis to the entire cohort, combining MGRS-A and MGRS-NA. We found that overall survival was influenced by inflammatory status (measured by CRP), hematologic disease burden (assessed by lactate dehydrogenase and hemoglobin levels), and ECOG performance status (a functional measure of patient frailty) at diagnosis. These findings emphasize the importance of a comprehensive assessment beyond hematologic parameters to evaluate risk and select the most suitable therapeutic approach. An accurate definition of each MGRS subtype, particularly distinguishing localized from systemic forms, appears essential to determine mortality risk profiles and guide personalized clinical management.

While our study provides valuable insights, its limitations stem from its retrospective design and relatively small sample size. Additionally, the high heterogeneity of histological patterns in MGRS-NA patients and the substantial lack of the ultrastructural analysis further constrain our ability to establish definitive causal relationships between clinical, histological, and laboratory parameters and the observed outcomes.

Additionally, the small number of events precluded more extensive statistical analyses, such as multivariate analysis. Extending the follow-up period could allow for a more detailed assessment of long-term outcomes across histological subgroups and therapeutic regimens. Advances in diagnostic techniques, including radiomics and DNA-based methods such as cell-free DNA analysis, could enable earlier detection and better characterization of MGRS, facilitating the design of prospective trials with larger cohorts to validate our findings [22–24]. Furthermore, the impact of novel tailored therapies, including anti-CD38 and anti-BCMA strategies, warrants evaluation to fully understand the effects of clone-directed therapies on MGRS outcomes [25]. Investigating immune cell compartments may also reveal potential roles for T-cell redirecting agents, such as CAR-T cells and bispecific antibodies, which have already transformed the treatment landscape for various hematologic malignancies [26]. Given the significant impact of kidney injury, a comprehensive management approach that combines supportive care with targeted treatments is essential for improving outcomes. To further enhance care, continued research is needed to refine diagnosis, identify prognostic factors, and develop standardized guidelines for the early recognition and management of MGRS, with a collaborative effort crucial advancing future studies.

## Supplementary Information

Below is the link to the electronic supplementary material.

Supplementary file1 (DOCX 20 kb)

## Data Availability

The datasets used and/or analyzed during the current study are available from the corresponding author on reasonable request.
