# Supplementary material for: Monoclonal gammopathy of renal significance (MGRS): retrospective monocentric analysis of clinical outcomes and treatment strategies
Source: Clin Exp Med. 2025 Apr 15;25(1):118. doi: 10.1007/s10238-025-01646-7 (PMC12000252; doi:10.1007/s10238-025-01646-7)
Supplement: Supplementary file 1 — Supplementary file1 (DOCX 20 kb) [file 10238_2025_1646_MOESM1_ESM.docx]

# **MONOCLONAL GAMMOPATHY OF RENAL SIGNIFICANCE (MGRS): RETROSPECTIVE MONOCENTRIC ANALYSIS OF CLINICAL OUTCOMES AND TREATMENT STRATEGIES**

**SUPPLEMENTARY MATERIALS**

**Supplementary Table 1.**

**Histological patterns and lesions, with predominant immune deposits in MGRS-NA patients**

| **Patient** | **Histological diagnosis** | **Glomerular**  **lesions** | **Tubular**  **Lesions *** | **Vascular**  **Lesions §** | **Prevalent IF deposition** |
| --- | --- | --- | --- | --- | --- |
| #1 | PGNMID | MPGN-pattern | None | None | Glomerular IgG kappa |
| #2 | MIDD | Mesangial proliferation | None | None | Glomerular IgA lambda, C3 |
| # 3 | LCDD | None | Severe IFTA | None | Tubular lambda chain |
| #4 | LCDD | None | ATI  Mild IFTA | None | Tubular lambda chain |
| #5 | PGNMID | MPGN with extracapillary proliferation | None | None | Glomerular IgG kappa, C3 |
| #6 | TMA | None | Moderate IFTA | Intimal edema  onion skinning type | Glomerular IgM kappa |
| #7 | PGNMID | MPGN pattern | Mild IFTA | Severe arteriolar hyalinosis | Glomerular IgG kappa |
| #8 | C3GN | MPGN pattern | Mild IFTA | None | Glomerular C3, lambda |
| #9 | PGNMID | MPGN pattern | None | None | Glomerular IgM kappa, C3 |
| #10 | PGNMID | MPGN pattern  Diabetic lesions | None | None | Glomerular IgA lambda |
| #11 | PGNMID | MPGN pattern with extracapillary proliferation | Intratubular cast fractured and angulated | None | Glomerular and tubular IgG kappa |
| #12 | PGNMID | MPGN pattern | None | None | Glomerular IgG, C3 |
| #13 | PGNMID | MPGN pattern | None | None | Glomerular IgG lambda |
| #14 | PGNMID | MPGN pattern with extracapillary proliferation | None | Hyalinosis subocclusive and fibrinoid necrosis | Glomerular IgG and C3 |
| #15 | PGNMID | MPGN pattern | None | None | Glomerular IgG, and C3 |
| #16 | CRYOGLOBULINEMIA | MPGN pattern with intracapillary thrombi | None | None | Glomerular IgM, C3 in pseudothrombi |
| #17 | PGNMID | MPGN pattern | None | None | Glomerular IgM, C3 and lambda |

Abbreviations: IF: immunofluorescence, PGNMID: Proliferative glomerulonephritis with monoclonal IgG deposits, TMA: Thrombotic microangiopathy, MPGN: membranoproliferative, IFTA: Interstitial fibrosis and tubular atrophy, ATI: acute tubular injury, C3GN: C3 Glomerulonephritis.

*Mild tubular atrophy was excluded from tubular lesions, § Atherosclerotic lesions were excluded from vascular lesions.

**Supplementary Table 2.**

**Predominant immune deposits and distribution of amyloid deposits in MGRS-A patients**

|  |  | **Amyloid accumulation in kidney compartments *** | | |  |
| --- | --- | --- | --- | --- | --- |
| **Patient** | **Prevalent IF deposition** | **Glomerular** | **Tubulo-interstitial** | **Vascular** | **EM** |
| #1 | IgG kappa chain | **++** | **+** |  |  |
| #2 | Lambda chain  and thioflavin | ++ | ++ |  | Electrodense fibrillar and nodular deposits |
| #3 | Lambda chain | + | +++ |  |  |
| #4 | IgG lambda chain  and thioflavin | + |  | ++ | Randomly oriented mesangial fibrillar material in the form of evident nodules |
| #5 | Lambda chain  and thioflavin | ++ | ++ | ++ |  |
| #6 | Lambda chain  and thioflavin | + |  | ++ |  |
| #7 | IgM lambda chain  and thioflavin | ++ | + | ++ |  |
| #8 | Lambda chain and thioflavin | ++ | + | ++ |  |
| #9 | Lambda chain |  | +++ |  |  |
| #10 | IgG lambda chain | ++ | ++ | ++ |  |
| #11 | IgG lambda chain and thioflavin | ++ |  | + |  |
| #12 | IgG lambda chain | +++ | +++ | +++ |  |

Abbreviations: IF: immunofluorescence, EM, electron microscopy.

* as evaluated by Congo Red staining
